# Predictors of long-term prognosis in rheumatoid arthritis-related interstitial lung disease

**DOI:** 10.1038/s41598-022-13474-w

**Published:** 2022-06-08

**Authors:** Juan Chen, Yaqiong Chen, Dehao Liu, Yihua Lin, Lei Zhu, Shuli Song, Yudi Hu, Tao Liang, Yongliang Liu, Wei Liu, Lin Weng, Qiyuan Li, Shengxiang Ge, Dana P. Ascherman

**Affiliations:** 1 Department of Rheumatology, School of Medicine, The First Affiliated Hospital of Xiamen University, Xiamen University, ZhenHai Road No.55, Xiamen, 361003 China; 2 Department of Radiology, School of Medicine, The First Affiliated Hospital of Xiamen University, Xiamen University, Xiamen, 361003 China; 3 Department of Respiratory Medicine, School of Medicine, The First Affiliated Hospital of Xiamen University, Xiamen University, Xiamen, 361003 China; 4 Division of Rheumatology and Clinical Immunology, University of Pittsburgh School of Medicine, Pittsburgh, 15261 USA; 5 School of Medicine, Xiamen University, Xiamen, 361104 China; 6 School of Life Sciences, Xiamen University, Xiamen, 361104 China; 7 State Key Laboratory of Molecular Vaccinology and Molecular Diagnostics, School of Public Health, Xiamen University, Xiamen, 361104 China; 8 Department of Pediatrics, School of Medicine, The First Affiliated Hospital of Xiamen University, National Institute of Data Science in Health and Medicine, Xiamen University, Xiamen, 361003 China

**Keywords:** Rheumatic diseases, Rheumatology

## Abstract

The aim of the study was to identify specific clinical and serum protein biomarkers that are associated with longitudinal outcome of RA-associated interstitial lung disease (RA-ILD). 60 RA patients with clinical and serological profiles were assessed by HRCT and pulmonary function tests (PFTs) at baseline (Year 0) and 5 years post enrollment (Year 5). Progression versus non-progression was defined based on changes in Quantitative Modified HRCT scores and PFTs over time. Specific serum protein biomarkers were assessed in serum samples at baseline and Year 5 by Multiplex enzyme-linked immunosorbent assays (ELISAs). At Year 5, 32% of patients demonstrated progressive RA-ILD, 35% were stable, and 33% improved. Baseline age and rheumatoid factor (RF) were significantly different between RA-ILD outcomes of progression vs. no-progression (*p* < 0.05). Changes in levels of CXCL11/I-TAC and MMP13 over 5 years also distinguished pulmonary outcomes (*p* < 0.05). A final binary logistic regression model revealed that baseline age and changes in serum MMP13 as well as CXCL11/I-TAC were associated with RA-ILD progression at Year 5 (*p* < 0.01), with an AUC of 0.7772. Collectively, these analyses demonstrated that baseline clinical variables (age, RF) and shifts in levels of selected serum proteins (CXCL11/I-TAC, MMP13) were strongly linked to RA-ILD outcome over time.

## Introduction

Rheumatoid arthritis (RA) may cause a variety of extra-articular manifestations, including interstitial lung disease (ILD)^1,2^. In the United States, the prevalence of RA-ILD ranged from 3.2 to 6.0 cases per 100,000 people from 2003 to 2014^3^. Equally important, a number of studies have indicated that RA-ILD causes decreased lung function and is a significant contributor to morbidity and mortality^3–6^. In fact, pulmonary complications are directly responsible for 10–20% of mortality in RA, corresponding to an estimated standardized mortality ratio of 2.5–5.0 compared with control populations^7^. Within 5 years of index date, about one-third of RA-ILD patients died based on Social Security Administration Death Index (SSDI) data^3^. In another study, the 5-year mortality of RA patients with and without ILD was 39.0% and 18.2%, respectively^8^.

Given the previously discussed morbidity/mortality of RA-ILD, it is necessary to find methods to not only make an early diagnosis, but also to reliably predict disease progression—particularly in individuals with subclinical and/or radiographically mild disease. Identifying baseline characteristics and longitudinal markers of disease progression would improve prognostication and help guide management of these patients^9^. Demonstrating the feasibility of this approach, previous cross-sectional studies have identified a number of serum protein biomarkers that are associated with the presence of ILD in RA patients, many of which are also associated with idiopathic pulmonary fibrosis (IPF)^9–14^. However, the ability of these and other biomarkers that focus on biological pathways involved in RA-ILD pathogenesis to *predict disease course* has not been studied^14,15^. Such biomarkers could be used to inform prognosis, suggest potential treatment options, and identify new areas of focus for drug discovery^15^. In this study, we therefore sought to identify specific clinical and proteomic biomarkers associated with longitudinal outcomes in RA-ILD.

## Results

### Baseline demographic and radiographic characteristics of the longitudinal cohort

**Figure 1:**
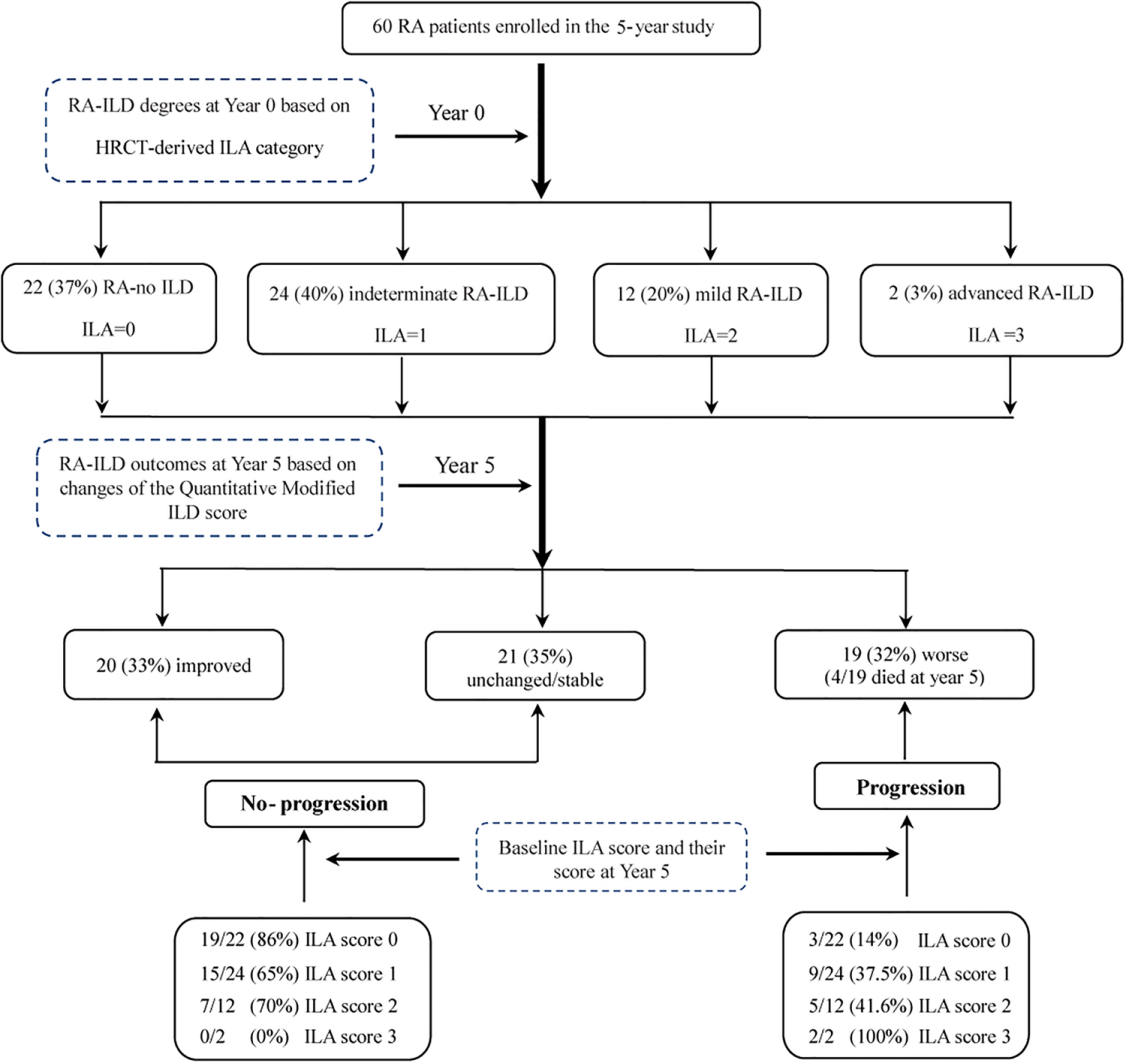
Study enrollment at Year 0 and Year 5. A flow diagram of the study shows the different degrees of RA-ILD based on 0–3 ILA scores in HRCT at Year 0 and outcome at Year 5 based on changes of Quantitative Modified ILD scores. Changes in the Quantitative Modified ILD score between 0–10% at Year 0 and Year 5 were classified as stable, while reduction of ILD scores by greater than 10% was classified as better (score reduction > 10%) and changes more than 10% increased as worse. HRCT = high-resolution computed tomography; ILD = interstitial lung disease; ILA = interstitial lung abnormality; RA = rheumatoid arthritis.

Of the 133 RA patients consecutively enrolled in our baseline cohort^11^, 63 were lost to follow up based on geographical constraints, 10 patients declined follow up studies, and 60 were followed longitudinally for the 5-year period of this study. The longitudinal cohort consisted of predominantly female (80%) never-smokers (86.7%), and their baseline mean age was 47.6 ± 16.6 years (Table1). These clinical and demographic features resembled those of the baseline inception cohort (data not shown), indicating that the 60 patients with follow up data were representative of the overall cohort. 4/60 patients (6.7%) died due to progressive ILD and/or pneumonia, resulting in a 5-year survival rate of 93% (Fig. 1); one of these patients had radiographic evidence of advanced RA-ILD (UIP pattern) at baseline and 3/4 had associated diffusion capacities (DLco) ranging from 50 to 60% predicted^11^.

**Table 1:** Characteristics of 60 RA patients at baseline.

	RA-no ILD	RA-indeterminate ILD	RA-mild ILD	RA-advanced ILD
	(ILA score 0)	(ILA score 1)	(ILA score 2)	(ILA score 3)
	(n = 22)	(n = 24)	(n = 12)§	(n = 2)Ŧ
**Demographic parameters**				
Age, years, mean ± SD	38.9 ± 16	51.4 ± 14.8*	62.2 ± 7.7***	75.5 ± 0.7*
Female, no. (%)	18 (82)	21 (88)	9 (75)	0 (0)
Smoker, no. (%)				
Never	19 (86)	22 (92)	11 (92)	0 (0)
Ever	3 (14)	2 (8)	1 (8)	2 (100)
Current	1 (5)	0 (0)	1 (8)	0 (0)
Pack-years of smoking, mean ± SD	1.1 ± 5.3	1.7 ± 5.6	2.5 ± 8.7	10.0 ± 14.1
Pack-years of secondhand smoking, mean ± SD	0 ± 0	1.0 ± 3.6	0.9 ± 2.9	10.0 ± 14.1
**RA parameters, median (IQR)**				
RF, IU/ml	47.5 (27.8–147.0)	191.5 (79.9–357.5)	324.5 (91.8–666.5)	905.5(538.2–1272.8)
Anti-CCP, units/ml	86.9 (54.3–290.4)	291.5 (62.1- 414.6)	480.1 (253–500.0)	351.7 (277.6–425.9)
DAS28	4.0 (2.4–5.1)	4.2 (3.4–5.2)	4.4 (3.7–5.2)	3.5 (3.2–3.9)
Duration of RA, years	3.5 (1.2–7.5)	2.0 (0.7–10)	5.5 (1.4–10)	2.0 (1.5–2.5)
**Medication use (ever) at baseline, no. (%)**				
Methotrexate	22 (100)	24 (100)	12 (100)	2 (100)
Leflunomide	10 (45)	9 (38)	4 (33)	0 (0)
Corticosteroid	2 (9)	5 (21)	2 (17)	1 (50)
Biologic	2 (9)	1 (4)	0 (0)	0 (0)
**Respiratory parameters, no. (%)**				
Cough	0 (0)	1 (4)	2 (17)	1 (50)
Dyspnea	0 (0)	1 (4)	2 (17)	1 (50)
**Spirometric parameters, median (IQR)**				
FEV1, percent of predicted	83.0 (76.5–95.5)	86.0 (76.5–90.0)	79.0 (65.0–87.2)	79.5 (66.8–92.2)
FVC, percent of predicted	79.0 (75.5–93.5)	83.0 (76.0–89.0)	73.0 (67.5–78.5)	86 (71.5–100.5)
Dlco, percent of predicted	86.0 (76.5–104.0)	94.0 (68.0–101)	60.0 (52.5–69.5)**	59 (56.5–61.5)
**Quantitative Modified ILD scoring in HRCT, median (IQR)**				
	0.8 (0.0–1.0)	2.0 (1.4–4.0) ***	8.0 (4.2–12.1)***	12.5 (12.2–12.8)*

RF = rheumatoid factor; anti-CCP = anti–cyclic citrullinated peptide; DAS28 = 28-joint Disease Activity Score. IQR = interquartile range. Age was significantly different among the subgroups: RA-mild ILD (ILA score2) and RA-no ILD (ILA score 0) *(p* < 0.001), RA–advanced ILD (ILA score3) and RA-no-ILD (ILA score0) (*p* < 0.05). The Quantitative Modified ILD scores in HRCT in RA-indeterminate ILD, RA-mild ILD and RA-advanced ILD groups were significantly different than RA-no ILD (*p* < 0.001, 0.001 and 0.05, respectively).

^**§**^n = 1 patient with UIP.

^**Ŧ**^n = 2 patients with UIP.

*p < 0.05, ** *p* < 0.01, *** *p* < 0.001 when compared with RA-no ILD at baseline; two sample t-test, Mann–Whitney U test (continuous variables) or Chi-square/Fisher’s exact test (categorical variables) were used to calculate p-values.

At baseline, the 60 patients from our initial cohort had varying degrees of parenchymal lung abnormalities (paralleling the distribution of radiographic severity in the larger baseline cohort^11^) and were subclassified as RA-no ILD (ILA = 0) in 22 (37%), RA-indeterminate ILD (ILA = 1) in 24 (40%), RA-mild ILD (ILA = 2) in 12 (20%), and RA-moderate/severe ILD (ILA = 3) in 2 (3%) patients (Table 1). As shown in Table 1, Quantitative Modified ILD scores corresponding to subcategories of indeterminate, mild, and advanced ILD differed significantly from those associated with RA-no ILD (*p* < 0.001, 0.001, and 0.05 respectively). Review of Table 1 also indicates that among baseline clinical and demographic variables, age differed significantly between ILA subgroups.

### Relationship between baseline ILD-specific variables and longitudinal outcomes

When longitudinal outcome was evaluated by Quantitative Modified ILD scoring, 19 patients (including the 4 patients who died) (32%) developed worsening ILD independent of pneumonia or fluid overload, 21 patients (35%) remained unchanged/stable, and 20 patients (33%) improved (Fig. 1). Comparative statistical analysis indicates that the Quantitative Modified ILD scoring system was more sensitive to changes in radiographic status that did not necessarily result in a shift in ILA subcategory. Despite this enhanced sensitivity for change in radiographic severity, *baseline* Quantitative Modified ILD scores were only slightly higher in progressors versus non-progressors (2.0 versus 1.5, *p* < 0.05) and therefore not predictive of worsening RA-ILD (Table 2).

**Table 2:** Baseline parameters and disease outcome at Year 5.

Baseline parameters	Outcome at Year 5	
	Progression	No-progression
	(Worse)	(Unchanged/Stable + Improved)
	(n = 19)	(n = 41)
**Demographic parameters**		
ILA score, no		
ILA score 0	3	19
ILA score 1	9	15
ILA score 2	5	7
ILA score 3	2	0
Age, years, mean ± SD	61.1 ± 16.0**	45.1 ± 16.1
Female, no. (%)	13 (68)	35 (85)
Pack-years of smoking, mean ± SD	3.7 ± 9.0	1.6 ± 7.3
Pack-years of secondhand smoking, mean ± SD	2.1 ± 9.2	1.8 ± 5.2
**RA parameters, median (IQR)**		
RF, IU/ml	195.0 (122.5–563)*	105.0 (27.4–300.0)
Anti-CCP, units/ml	255.0 (81.1–448.1)	274.8 (61.1–500.0)
DAS28	4.2 (3.3–5)	4.1 (3–5.2)
Duration of RA, years	3.0 (1.0–10.0)	2.0 (1.0–9.0)
**Cumulative dosages of medications used over 5-year (mg), median (IQR)§**		
Methotrexate	260.0 (51.2–390.0)*	325.0 (260.0–390.0)
Leflunomide	1825 (1825.0 – 3650.0)	1825 (1825.0–1825.0)
Corticosteroid	157.5 (13.1–681.2	912.5 (453.1–912.5)
**Respiratory parameters, no. (%)**		
Cough	4 (21)*	0 (0)
Dyspnea	4 (21)*	0 (0)
**Spirometric parameters, median (IQR)**		
FEV1, percent of predicted	79.0 (65.2–89.2)	82.0 (76.2–93.0)
FVC, percent of predicted	75.5 (62.0–84.0)	79.0 (75.5–92.5)
DLco, percent of predicted	58.0 (52.5–70.5)**	86.0 (69–101.8)
**HRCT abnormalities, no. (%)**		
UIP pattern	3 (16)*	0 (0)
Non-UIP pattern	16 (84)	41 (100)
**Quantitative Modified ILD scoring in HRCT, median (IQR)**		
	2.0 (1.0–10.0)*	1.5 (0.0–3.0)

RA-ILD outcomes at Year 5 were defined as progression versus no-progression based on changes of the Quantitative Modified ILD HRCT scores from Year 0 to Year 5. Unchanged/stable (≤ 10% change) and improved (≥ 10% decrease) in the Quantitative Modified ILD scores were designated as no-progression, while worsening scores (> 10% increase in Quantitative Modified ILD scores) were classified as progression. Among the 56 patients who were alive at Year 5, 39 had available PFT data from both Year 0 and Year 5 time points. Baseline age, RF, cough, dyspnea, DLco% and non-UIP HRCT pattern in the progression group were significantly different compared to the no-progression group (*p* < 0.01, 0.05, 0.05, 0.01, 0.05, 0.05, respectively). The cumulative dosage of methotrexate (mg) used over 5-years in the progression group was significantly lower than in the no-progression group (*p* = 0.027).

^**§**^The percentages of patients using methotrexate, leflunomide and corticosteroid in the progression group were 100%, 42%, and 21%, respectively. The percentages of patients using methotrexate, leflunomide and corticosteroid in the no-progression group were 100%, 53% and 15%, respectively. Median (IQR) values for cumulative medication dose were assessed across entire subgroups (progressors vs. non-progressors), but only encompassed those individuals who used the specified medication during the 5 year study period.

* *p* < 0.05, ** *p* < 0.01, *** *p* < 0.001, progression group versus non-progression group respectively. *p*-values were derived from two samples t-test/Mann–Whitney U test (continuous variables) and Chi-square /Fisher’s exact test (categorical variables).

#### Baseline HRCT

As demonstrated in Fig. 1, the risk of disease progression (as assessed by Quantitative Modified ILD scores) varied by baseline ILA category. While only 14% (3/22) of individuals initially classified as ILA = 0 (no ILD) at baseline developed evidence of parenchymal lung abnormalities consistent with ILD, 37.5% (9/24) of those with indeterminate ILD at baseline (ILA = 1) had evidence of progressive ILD at Year 5. Among individuals with ILA = 2 (mild ILD) and ILA = 3 (moderate/severe ILD) at baseline, 50% (7/14) had worsening Quantitative Modified ILD scores over the 5 year time period of this study.

To further explore the relationship between baseline HRCT characteristics and risk of disease progression, we assessed the correlation between *patterns* of HRCT abnormalities at baseline and disease outcome. While 16% of patients (n = 3, consisting of ILA = 2 (n = 1) and ILA = 3 (n = 2)) in the progression group had evidence of UIP at baseline (versus 84% non-UIP, n = 16), none of the patients in the no-progression group had UIP at baseline (Table 2).

#### PFTs

The associations between baseline pulmonary function test parameters and outcomes determined by Quantitative Modified ILD HRCT scores were analyzed (Table 2). Baseline percent predicted DLCO (DLco%) in the progression group was significantly lower than in the no-progression group (*p* < 0.01). However, no significant differences in baseline percent predicted FVC (FVC%) or FEV1 (FEV1%) separated the progression versus no-progression groups (*p* > 0.05) (Table 2). Among the 56 patients who were alive at Year 5, 39 had available PFT data from both Year 0 and Year 5 time points. While 7 patients (18%) became worse based on defined FVC criteria, 19 (49%) remained unchanged/stable, and 13 (33%) improved (data not shown). Correlation with HRCT-based determination of disease progression was relatively strong, as 28/39 (72%) patients undergoing both PFTs and HRCT at study entry and Year 5 were similarly classified as progressors versus non-progressors whether disease course was assessed by changes in FVC% or changes in radiographic ILD scores.

### Baseline clinical risk factors, medication usage, and longitudinal outcomes

As shown in Table 2, the relationships between baseline clinical parameters (age, sex, smoking history, RA disease duration, articular disease activity (DAS28), RF, and anti-CCP) and long-term outcomes based on changes of the Quantitative Modified ILD HRCT scores over five years were analyzed. Baseline age in the progression group was significantly higher than baseline age in the no-progression group (*p* < 0.01). Similarly, baseline titers of RF in the progression group were significantly higher than baseline titers of RF in the no-progression group (*p* < 0.05) (Table 2). Of note, pack-years of smoking in the progression group were higher (3.7 ± 9.0) than in the no-progression group (1.6 ± 7.3), though this did not reach statistical significance; (*p* > 0.05). Other parameters, including baseline sex, anti-CCP antibodies, and articular disease activity (measured by DAS28) were not different between the progression and no-progression groups (*p* > 0.05). Finally, four patients (6.7%) with symptoms of cough and/or dyspnea were in the progression outcome group, while none of the patients in the no progression group had these symptoms—a difference that was statistically significant (*p* < 0.01, Table 2).

#### Medications used

There was a statistically significant association between cumulative dosages of methotrexate (MTX) used and outcome measured by the Quantitative Modified ILD HRCT scores, as the cumulative dosage of methotrexate used in the progression group was significantly lower than that in the no-progression group over the 5 year period of this study (*p*<0.05) (Table 2). However, when the 3 patients with baseline UIP were excluded from this analysis, the cumulative dose of methotrexate no longer differed between progressors and non-progressors (325 (260–390) mg in progressors versus 292.5 (208.1–422.5) mg in non-progressors, p>0.05). Similarly, there were no significant associations between 5 year cumulative dosages of leflunomide or prednisone and ILD outcomes (Table 2).

### Serum levels of biomarkers and longitudinal outcomes

To determine serum biomarkers that might be associated with the long-term prognosis of RA-ILD, we measured serum levels of 36 proteins consisting of cytokines, chemokines, growth factors, and remodeling proteins (MMPs) that have previously been assessed in patients with IPF as well as RA-ILD^16,17^. We then analyzed the relationship between serum levels of these serum biomarkers (both baseline and change over time) and outcomes. As shown in Supplementary Fig. 2 and 3, none of the assessed protein levels (*at baseline*) were associated with RA-ILD outcome (progression versus no-progression) measured by shifts in the FVC%, DLco%, or Quantitative Modified ILD HRCT scores from Year 0 to Year 5 (*p* > 0.05).

**Figure 2:**
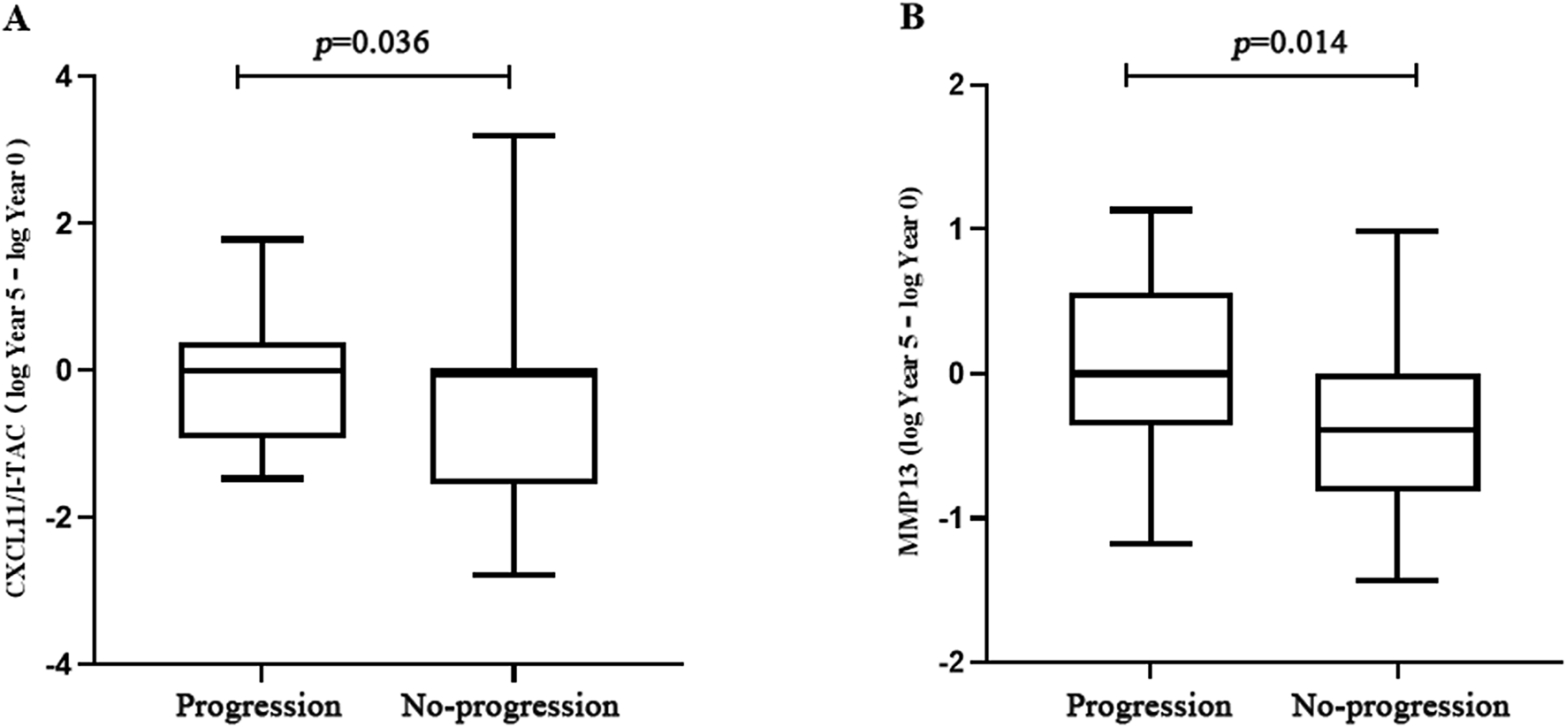
Relationship between changes in serum levels of biomarkers from Year 0 to Year 5 and radiographic outcome. Box plots demonstrate that serum levels of A) CXCL11/I-TAC and B) MMP-13 from Year 0 to Year 5 (log Year 5—log Year 0) (y-axis) were significantly associated with RA-ILD outcomes (progression versus no-progression, x-axis), as assessed by changes in Quantitative Modified ILD scores (*p* = 0.036 and *p* = 0.014 for CXCL11/ITAC and MMP-13, respectively). Horizontal bars signify median values.

As a complementary approach to identify markers of RA-ILD progression, we assessed the correlation between *changes* in serum levels of selected cytokines/chemokines/MMPs and RA-ILD outcomes measured by PFT parameters as well as Quantitative Modified ILD HRCT scores. As shown in Fig. 2, changes in serum levels of CXCL11 and MMP-13 from Year 0 to Year 5 (log Year 5—log Year 0) were significantly associated with different outcomes (progression versus no-progression) measured by Quantitative Modified HRCT scores in the 56 patients (excludes n = 4 patients who died prior to study completion) who had serum samples at baseline and Year 5 time points (*p* < 0.05). However, levels of these serum proteins were not statistically associated with different outcomes measured by PFT parameters. Correlations between shifts in the serum levels of other defined protein biomarkers and radiographic/PFT outcomes between Year 0 and Year 5 were not statistically significant and are reported in Supplementary Fig. 4.

### Factors predicting progression of RA-ILD

**Figure 3:**
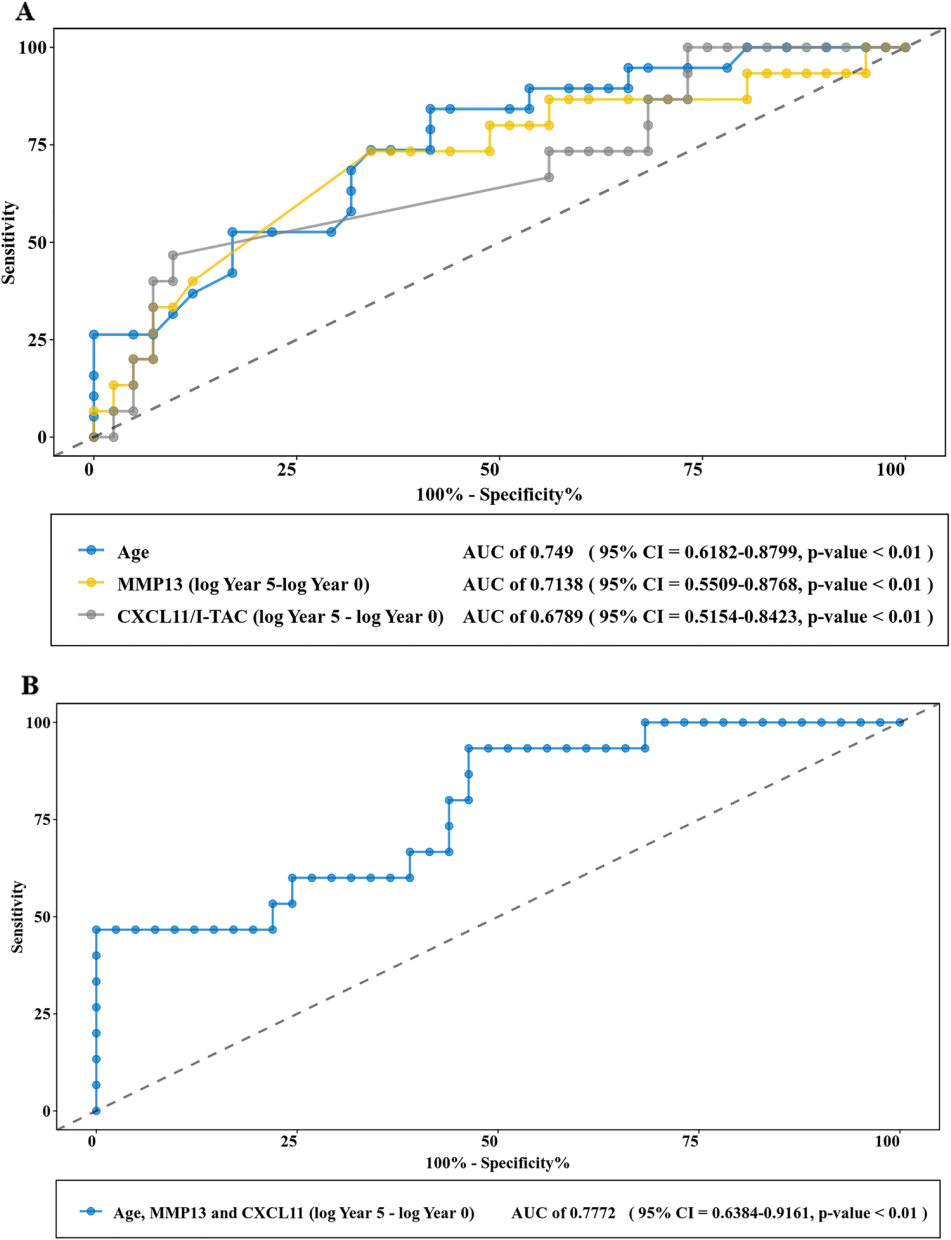
ROC analyses of disease prediction models. Age and changes in serum levels of MMP13 (log Year 5-log Year 0) as well as CXCL11 (log Year 5-log Year 0) over 5-years were fit into the final logistic regression model. ROC analyses for models based on individual parameters of age, changes in MMP13 level, or changes in CXCL11 level (3a) versus the combination of age, changes in MMP13 level, and changes in CXCL11 level reflect variables associated with the risk of progression in RA-ILD (as measured by Quantitative Modified ILD scores). The respective AUCs for these models are 0.749 (age), 0.7138 (change in MMP-13), 0.6789 (change in CXCL11) and 0.7772 (combined variables)—all with *p* < 0.01.

More detailed statistical analyses were conducted to see which combination of demographic variables, clinical risk factors (RF, anti-CCP, DSA28 and disease duration at baseline), and serum proteins (shifts in CXCL9/MIG, CXCL10/IP10, CXCL11/I-TAC and MMP13 levels over 5 years) were associated with binary outcomes of progression versus no-progression. Significant differences were found between the progression and no-progression groups in age, RF, and changes (log Year 5—log Year 0) in serum levels of CXCL11/I-TAC and MMP13 (*p* < 0.05, Table 3). We first used the Spearman coefficient for continuous variables to ensure an r value < 0.5 for each of these parameters in order to avoid multicollinearity. Based on these calculations and assessment of AIC values, age and change in MMP13 as well as CXCL11 levels were retained in the final logistic regression model (Table 4). Area under the curve derived from ROC analysis was 0.7772 (95% confidence interval: 0.6384–0.9161, *p* < *0.01*), indicating that the model incorporating age and change in both MMP13 and CXCL11 levels effectively distinguished disease progressors from non-progressors (Fig. 3).

**Table 3:** Factors predicting radiographic progression in RA-ILD.

	Progression (n = 19)	No-progression (n = 41)	*p*-value
Age, years	61.1 ± 16.0	45.1 ± 16.1	**0.002**
RF, IU/ml	195.0 (122.5–563)*	105.0 (27.4–300.0)	**0.044**
Anti-CCP, untis/ml	255.0 (81.1–448.1)	274.8 (61.1–500.0)	0.904
DAS28	4.2 (3.3–5)	4.1 (3–5.2)	0.994
Duration of RA, years	3.0 (1.0–10.0)	2.0 (1.0–9.0)	0.542
CXCL9/MIG (log Year 5–log Year 0) *	−0.1 (−0.8–0.3)	−0.3 (−0.7–0.0)	0.215
CXCL10/IP10 (log Year 5–log Year 0) *	−0.8 [−1.3–0.0]	−0.6 [−0.9–(−0.3)]	0.667
CXCL11/I-TAC (log Year 5–log Year 0) *	0.0 [−0.9–0.4]	0.0 (−1.5–0.0)	**0.036**
MMP13 (log Year 5–log Year 0) *	0.0 (−0.4–0.6)	−0.4 (−0.8–0.0)	**0.014**

* 56 patients had cytokine analysis at Year 5 (excludes n = 4 progressors who died prior to Year 5 serum collection).

After normality testing (K-S test) for the nine indices, age conformed to a normal distribution. The actual values were presented as mean ± standard deviation. RF, anti-CCP, DSA28 and disease duration at baseline did not adhere to a normal distribution and were therefore converted by logarithm transformation and presented as median values (interquartile range). Changes in cytokine levels of CXCL11/I-TAC and MMP-13 from Year 0 to Year 5 were converted by logarithm transformation and expressed as (log Year 5—log Year 0). Significant differences were found between the progression and the no-progression groups for age, RF, CXCL11/I-TAC (log Year 5—log Year 0) and MMP13 (log Year 5—log Year 0). Other variables did not show statistically significant differences.

**Table 4:** Relationship between selected risk factors and progression of RA-ILD.

	beta coefficient	*P*-value
Age	0.057	0.015
MMP13 (log Year 5—log Year 0)	1.072	0.156
CXCL11 (log Year 5—log Year 0)	0.253	0.509

Descriptive analyses showed differences in risk of RA-ILD progression for age, RF, and changes (log Year 5-log Year 0) in serum levels of CXCL11/ITAC and MMP13. We conducted Spearman correlation analyses for continuous variables to exclude highly correlated variables (r > 0.5 and *p* < 0.05) from the final logistic regression model in order to avoid multicollinearity. Based on these considerations and impact on AIC values, age, MMP-13 (log Year 5-log Year 0), and CXCL11 (log Year 5-log Year 0) were selected to include in the final model.

## Discussion

This study demonstrated that several clinical/demographic variables and serum protein biomarkers were associated with the long-term prognosis of RA-ILD. While changes in serum levels of CXCL11 and MMP-13 were significantly associated with longitudinal progression of RA-ILD in univariate analyses, baseline age and RF were also associated with the long-term outcomes of RA-ILD. Our final binary logistic regression model revealed that baseline age and changes in serum levels of MMP13 as well as CXCL11 were associated with RA-ILD progression (Yes vs. No) at Year 5.

Of note, radiographic disease progression was accompanied by increased levels of CXCL11/I-TAC and MMP13 over this time period, indicating that these serum proteins could serve as prognostic biomarkers in our longitudinal cohort. However, the basis for these associations remains unclear. Furthermore, it is unclear why some biomarkers relate to disease activity at a specific time point, while other markers predict disease progression. Previous cross-sectional studies have shown, for example, that a number of serum protein biomarkers are associated with the presence of both mild and advanced forms of RA-ILD—including MMP-7 and IP-10/CXCL10^11,12,14,18,19^—suggesting that these mediators contribute to the pathophysiology of RA-ILD. Moreover, a 7-biomarker signature consisting of MMP-1, MMP-2, MMP-7, MMP-9, IL-1 receptor antagonist, soluble CD40L, and CXCL9 effectively differentiated RA-ILD from RA–no ILD with high sensitivity and specificity in a separate cohort, yielding an AUC of 0.93^10^.

We have extended these findings by showing that CXCR3-binding chemokines such as CXCL11/I-TAC predict radiographic progression of RA-ILD, suggesting that pro-inflammatory disease pathways and TH1 skewing promoted by this chemokine contribute to progression of ILD. On the other hand, the demonstrated relationship between shifting MMP13 levels and disease outcome points to an important role for tissue remodeling in progression of RA-ILD.

Our present study indicated that older age was a clinical risk factor associated with worse long-term RA-ILD outcomes, correlating positively with the risk of progressive RA-ILD. These findings dovetail with other studies demonstrating that older age and male sex are associated with less favorable long term outcome^15,20–25^. In fact, one study has shown that for every 10 year increase in age, the risk of ILD increased by 59.9% in RA^26^.

In addition to age, baseline RF titers were also associated with RA-ILD outcome in our study—though we did not observe similar associations with baseline levels of anti-CCP antibodies. Although the literature does support some correlation between the levels of anti-CCP/anti-CCP2 antibodies and the likelihood of RA-ILD^10,27,28^, our failure to see any statistical correlation with disease outcome could reflect the absence of lung-specific antigens in CCP ELISAs.

Apart from these biomarker analyses, a novel observation emerging from our study was that the prognosis of indeterminate/mild RA-ILD (ILA 1–2) was relatively favorable over the 5 year period of this study. In fact, 45% (16/36) of RA-ILD patients in these subcategories improved over this time period based on HRCT criteria, and 29% (6/21) of patients improved according to changes of FVC. One plausible explanation for the discrepancy between our findings and previous studies demonstrating much worse outcome^8,15,28^ is that a substantial portion of our RA cohort had less severe ILD at study entry (37% no-ILD, 40% indeterminate ILD, and 20% mild/early ILD). Comparing baseline ILD scoring and outcome in our study demonstrated that more individuals with baseline ILA scores of 0, 1, and 2 were in the no-progression group (41/58 individuals with ILA 0–2 did not progress) relative to those individuals with a baseline ILA score of 3 (where both patients progressed). Therefore, RA patients with less severe stages of ILD (e.g., indeterminate/mild ILD) at baseline are less likely to progress—though our data do not allow us to conclude whether this trend reflects the natural history of mild/subclinical ILD or if these milder forms of RA-ILD are more responsive to immunomodulatory treatments targeting articular disease.

Beyond these considerations, another reason for generally favorable outcomes in this cohort could be that most of enrolled patients (80%) were female with very low primary smoking rates. In support of these hypotheses, our data show that the percentage of female RA-ILD patients was higher among non-progressors (85%) than in progressors (68%). On the other hand, pack-years of smoking in the progression group were higher (3.7 ± 9.0 pack-years) than in the no-progression group (1.6 ± 7.3 pack-years). Although not statistically significant (p > 0.05) due to the small number of smoking patients (5/60) in this study, the latter observation is consistent with previous studies indicating that cigarette smoking is a risk factor and an independent predictor of RA-ILD^9,10,29–31^.

In terms of baseline imaging characteristics, all 41 patients in the no-progression outcome group had HRCTs showing a non-UIP pattern with predominant bilateral, ground-glass opacities—contrasting with the progression group in which only 84% of patients demonstrated a non-UIP pattern. Distinguishing UIP from non-UIP patterns clearly has important prognostic implications for RA-ILD^4,32,33^, as the presence of radiological honeycombing may be a useful predictor of poor prognosis^4,20,33,34^. In fact, UIP-patterned ILD resembles idiopathic pulmonary fibrosis (IPF) and was the primary cause of death in RA-ILD^4,18^. On the other hand, idiopathic NSIP has a better prognosis and often responds to anti-inflammatory therapy^32^, suggesting that inflammatory cells likely to be present in non-UIP forms of RA-ILD might be responsive to immunosuppressive treatment^35^. In turn, because changes in the therapeutic approach to RA patients with ILD may alter the profile and outcome of this disease^4,34^, detection and management of RA-ILD patients at earlier stages with non-UIP HRCT patterns could prevent progression to more advanced stages of this devastating extra-articular disease complication.

Our study has several limitations. Importantly, this study was a single-center study with a relatively small number of patients in each subgroup of RA-ILD having follow-up data. Without additional patients in baseline subcategories of RA-no ILD (ILA = 0) and indeterminate/early ILD (ILA = 1 + ILA = 2), we were not able to specifically address important questions regarding risk factors for a) incident ILD or b) progression of indeterminate/early ILD to more advanced forms of fibrotic ILD. However, because one of the two patients with more severe ILD (ILA = 3) at baseline died prior to the 5 year endpoint of this study (and was therefore excluded from analysis of changes in serum protein biomarkers), our final logistic regression model does, in fact, represent risk factors for disease development/progression in RA patients with absent, indeterminate, or early/subclinical ILD. Beyond these issues, not every subject included in this study had PFT data available—and in cases where both PFTs and HRCT scans were performed, the level of agreement between these diagnostic modalities in measuring disease outcome (progression versus non-progression) was not perfect, with a concordance rate of 0.72. Given these and other limitations, longitudinal assessment of additional RA-ILD patients from larger, independent cohorts will be necessary to verify our findings and fully determine the predictive value of identified biomarkers, including their ability to distinguish outcomes of RA-ILD from those of other forms of ILD and/or IPF.

## Conclusions

While baseline age and RF predicted RA-ILD outcomes of progression versus no-progression, shifts in the level of CXCL11 and MMP-13 over 5 years were also correlated with long-term prognosis of RA-ILD. In multivariate analyses, baseline age as well as changes in levels of MMP-13 and CXCL11 were associated with the risk of progression of RA-ILD.

## Methods

### Patients

The study was designed as a 5-year, single-center, observational study that was conducted at the first affiliated Hospital of Xiamen University, School of Medicine, Xiamen University, China. Ethical approval for the study was obtained by the Ethical Committee of the first affiliated Hospital of Xiamen University, School of Medicine, Xiamen University (Approval number.KY2017-026). All experiments were performed in accordance with relevant guidelines and regulations.

133 adult patients with RA (> 18 years old) who met the American College of Rheumatology (ACR) 1987 criteria for definite RA^36^ were consecutively enrolled between July 2012 and March 2013^11^. Written informed consent was obtained from each participant. Of these patients, 60 were followed for a minimum of 5 years (63 lost to follow up, 10 declined follow up studies). Serum samples were collected at baseline (Year 0) and at 5-year study visits (Year 5). Clinical data were obtained within 3 days of serum sampling.

RA patient-specific variables consisting of baseline age, sex, disease duration, smoking history (never/current/former smoking as well as second-hand cigarette smoke exposure), articular disease activity (DAS28), titers of RF, and anti-CCP were recorded (Supplementary Fig. 1). Dyspnea was evaluated with the University of California, San Diego Shortness of Breath Questionnaire^37^. ILD-specific variables including high-resolution computed tomography scans (HRCT) and pulmonary function tests (PFTs) [forced vital capacity percent (FVC%), forced expiratory volume in 1 s (FEV1%) and lung diffusion for carbon monoxide (DLco%)] were measured. Selected biomarker variables, which included a range of cytokines, chemokines, acute-phase proteins, and MMPs potentially related to the mechanism of RA-ILD, were tested. All clinical, radiographic, PFT, and biomarker indices were collected at baseline and Year 5. Medical history and the use of disease-modifying anti-rheumatic drugs (DMARDs) and/or corticosteroids were obtained at these time points.

### Imaging and scoring

HRCT images were obtained from all patients at Year 0 and Year 5. A numerical score was assigned based on the type and distribution of interstitial lung abnormalities (ILA) consisting of septal lines, reticulation, traction bronchiectasis, cyst formation, and/or ground-glass attenuation, utilizing a published scale of ILA ranging from 0 to 3^11,38,39^. In terms of RA-ILD sub-classification, predominant bilateral, ground-glass opacities without honeycombing or architectural distortion were designated as a non-UIP pattern, while predominant honeycombing and traction bronchiectasis or architectural distortion were identified as a UIP pattern. Based on the extent and distribution of these parenchymal lung abnormalities, patients with a UIP pattern were classified as ILA = 2 (n = 1) or ILA = 3 (n = 2).

When Year 0 and Year 5 HRCT images from the same patient were compared, ILD severity was calculated using a more detailed Quantitative Modified ILD scoring system in which each lung was divided into three zones of upper, middle and lower at the level of the aortic arch and the left inferior pulmonary vein. In the Quantitative Modified ILD scoring system, the following HRCT findings were coded as present or absent in each zone: ground–glass opacity (GGO), consolidation /air space disease (AS), mixed GGO/AS, parenchymal micronodules (< 7 mm), reticular opacities, linear opacities or septal lines, peri-bronchovascular thickening, honeycombing, and traction bronchiectasis/bronchiolectasis. The severity of abnormalities in each of the defined lung zones was then graded as: 0 = no abnormality; 1 = trace/minor abnormality (< 5% area); 2 = mild abnormality (5–20% area); 3 = moderate abnormality (> 20–50% area); or 4 = severe abnormality (> 50%) (Supplementary Table 1). The maximum possible ILD score was 192 in this grading scheme.

Quantitative Modified ILD scores at Year 0 and at Year 5 for each patient were assessed by three independent reviewers who were blinded with regard to the clinical status of patients. Scans with discrepant readings were then evaluated and resolved by consensus.

### RA-ILD disease outcome at Year 5 based on HRCT and PFTs

RA-ILD radiographic status at Year 5 was defined as unchanged/stable, improved, or worse based on changes in the Quantitative Modified ILD HRCT score relative to ILD scores at Year 0. Changes in ILD score between 0–10% at Year 0 and Year 5 were classified as unchanged/stable, while reductions in ILD scores by greater than 10% were classified as improved (score reduction > 10%). Conversely, increases in ILD scores by more than 10% were classified as worse (score increase > 10%).

ILD disease outcome was also measured based on relative changes in functional parameters (FVC and DLco) between Year 0 and Year 5. While greater than 10% decreases in the ratio of Year 5/Year 0 FVC and/or DLco were defined as worse, greater than 10% increases in the ratio of Year 5/Year 0 FVC and/or DLco were classified as improved and less than 10% change in the Year 5/Year 0 FVC and/or DLco were considered unchanged/stable^40,41^.

### Multiplex ELISA

Multiplex ELISAs were performed using Luminex xMAP technology in a 96-well microplate format (eBioscience, Procarta). A combined 36-plex assay was used to determine serum levels of a range of cytokines, chemokines, acute-phase proteins, and MMPs (listed in Supplementary Table 2) potentially related to the mechanism of RA-ILD. Of note, serum samples at Year 0 and at Year 5 were assessed at the same time for all enrolled patients.

### Statistical analysis

All continuous variables were evaluated for normality to ensure uniformity in concentration and dispersion. Logarithm transformed values were used for variables not adhering to a normal distribution. While mean and standard deviation were presented for normally distributed variables, median and interquartile range (IQR) were presented for variables that did not follow a normal distribution.

Bivariate analyses were conducted using Chi-square or Fisher’s exact test for categorical variables and outcomes; alternatively, T tests or Mann–Whitney U tests were used for continuous variables (e.g., serum biomarker levels) and outcomes, and for comparing the characteristics of different groups of RA-ILD patients at baseline. To determine correlations among continuous variables and analyze the influence of confounding factors, Spearman correlation coefficients were calculated.

Additional analyses were conducted to see which demographic variables, clinical risk factors, or serum proteins were associated with disease progression. These variables were included in the final logistic regression model as candidate covariates to assess associations with the outcome of disease progression versus no progression. We further evaluated the performance of our classification model using receiver operating characteristic (ROC) analysis to calculate area under the curve (AUC) values.

All statistical analyses were performed using IBM SPSS Statistics, version 20.0. Two-sided p-values ≤ 0.05 were considered statistically significant.

## Supplementary Information

Supplementary Information.

## Data Availability

The datasets generated during and/or analyzed during the current study are available from the corresponding author on reasonable request.
