# Supplementary material for: Predictors of long-term prognosis in rheumatoid arthritis-related interstitial lung disease
Source: Sci Rep. 2022 Jun 8;12:9469. doi: 10.1038/s41598-022-13474-w (PMC9177673; doi:10.1038/s41598-022-13474-w)
Supplement: Supplementary file 1 — Supplementary Information. [file 41598_2022_13474_MOESM1_ESM.pdf]

Predictors of long-term prognosis in rheumatoid arthritis-related interstitial lung disease

Juan Chen[1 §*], Yaqiong Chen[1*], Dehao Liu[2], Yihua Lin[3], Lei Zhu[4], Shuli Song[1], Yudi Hu[5], Tao Liang[6], Yongliang Liu[7], Wei Liu[7], Lin Weng[1], Qiyuan Li[8], Shengxiang Ge[6], Dana P. Ascherman[4 §]

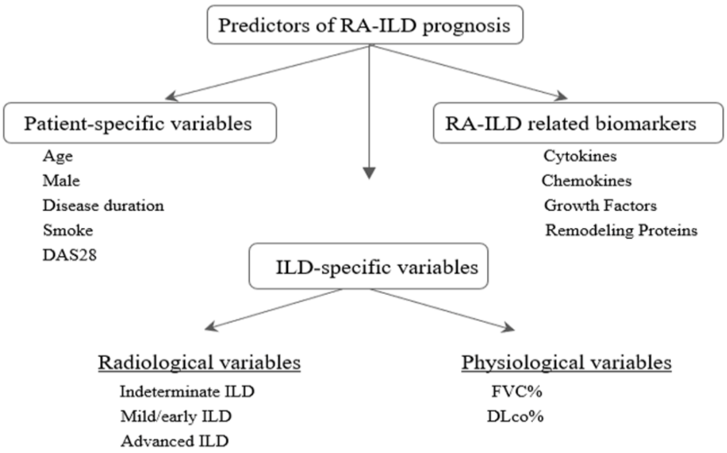

Supplementary Figure 1. Candidate predictors of RA-ILD progression**.** This figure outlines potential predictors of RA-ILD progression evaluated in unadjusted statistical analyses across our 5-year study. RF=rheumatoid factor; anti-CCP=anti–cyclic citrullinated peptide; DAS28=28-joint Disease Activity Score. HRCT= high-resolution computed tomography; FVC%=percent predicted, forced vital capacity; DLco%=percent predicted, lung diffusion for carbon monoxide; RA= rheumatoid arthritis; ILD= interstitial lung disease.

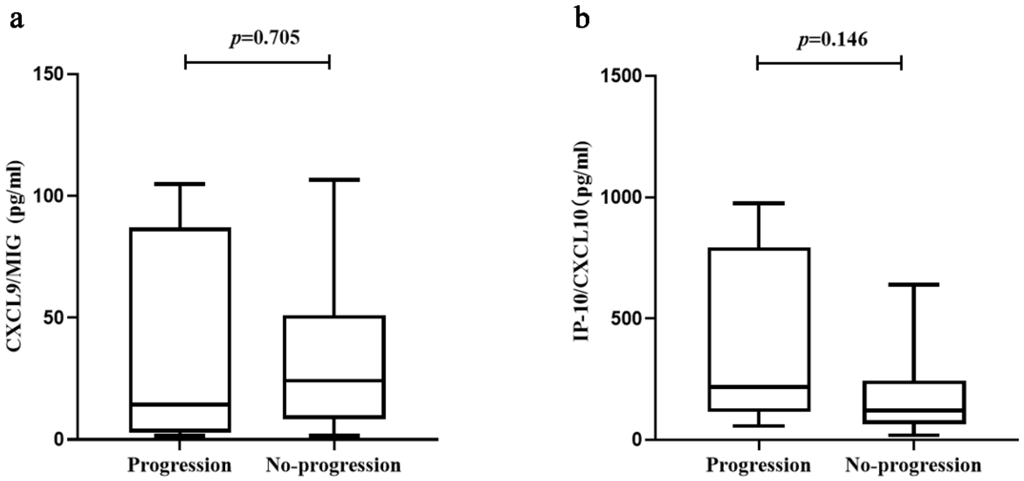

Supplementary Figure 2. Relationship between baseline serum levels of CXCL9/MIG, CXCL10/IP10, and outcome (progression versus no-progression) based on the changes of FVC% over 5 years. a-b. Serum levels of CXCL9/MIG and CXCL10/IP10 at baseline were not significantly different between the progression and no-progression groups based on the changes of FVC% from Year 0 to Year 5 (p=0.705, p=0.146, respectively).

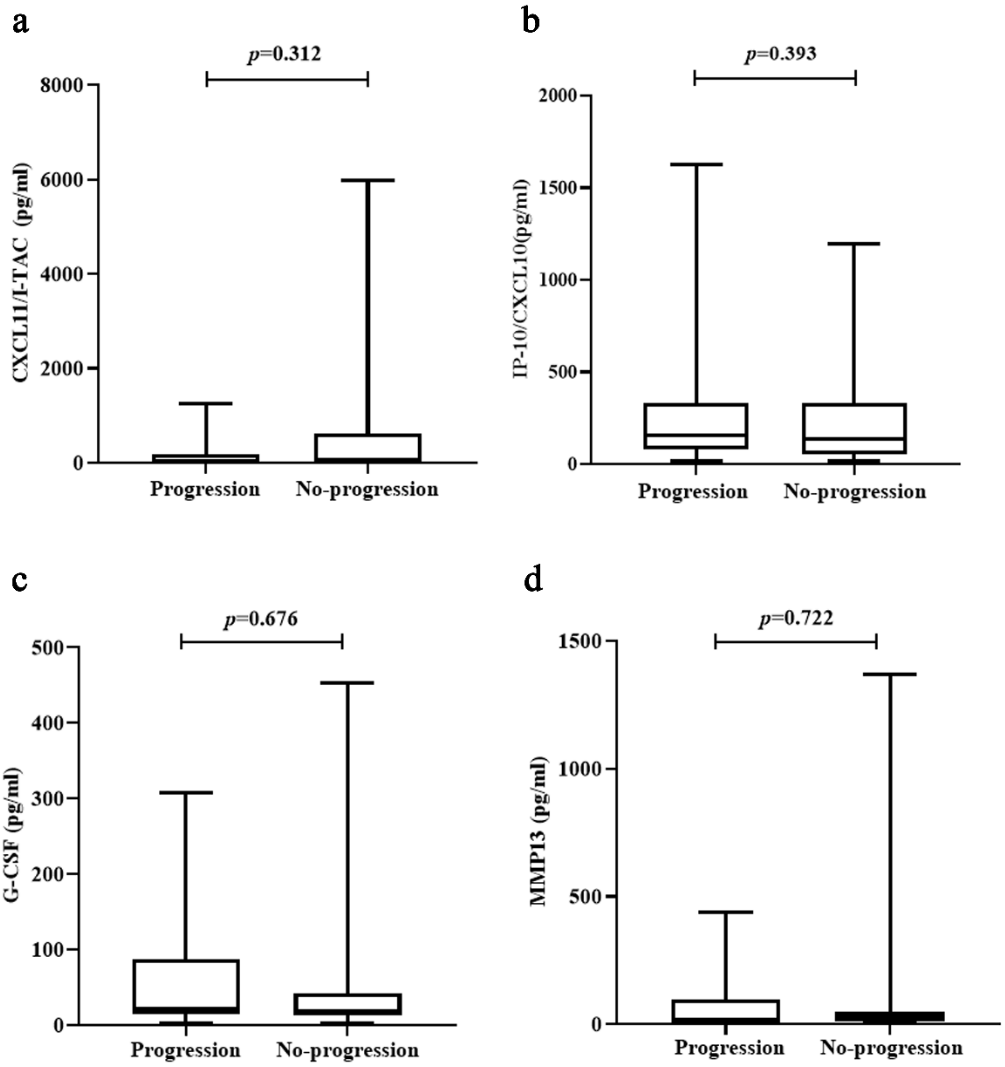

Supplementary Figure 3. Baseline serum levels of CXCL11/I-TAC, IP-10/CXCL10, G-CSF and MMP13 and outcome (progression vserus no-progression) measured by changes of DLco% over 5-year. a-d. Baseline serum levels of CXCL11/I-TAC, IP-10/CXCL10, G-CSF and MMP13 were not statistically significant differences between progression group and no-progression group based on the changes of DLco% from Year 0 to Year 5(p=0.312, p=0.393, p=0.676, p=0.722 respectively).

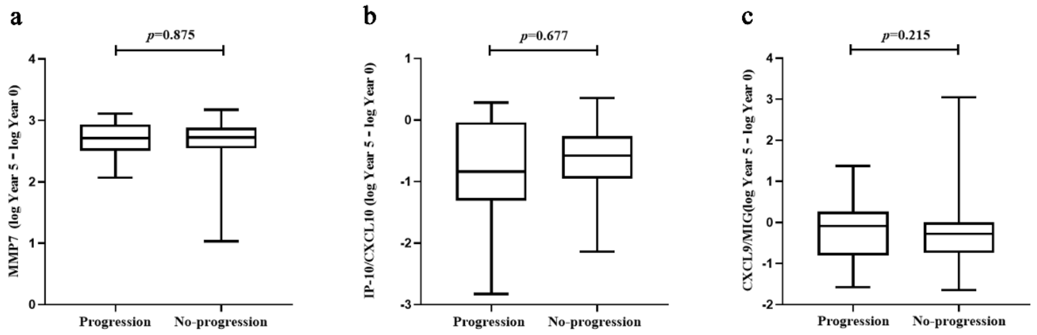

Supplementary Figure 4. Relationship between binary disease outcome and changes in serum levels of MMP7, CXCL10/IP10 and CXCL9/MIG from Year 0 to Year 5. Panels a-c assess the relationship between changes in serum levels of MMP7, CXCL10/IP10, and CXCL9/MIG from Year 0 to Year 5 (log Year 5-log Year 0) and disease outcome (progression versus no progression), as measured by the HRCT Quantitative Modified ILD scoring system. Respective *p*-values: 0.875, 0.677, and 0.215.

Supplementary Table 1. HRCT Quantitative Modified ILD scoring system

		Left Upper zone	Left Middle zone	Left Lower zone	Right Upper zone	Right Middle zone	Right Lower zone
	Abnormal (mark the abnormal zones)						
	**Radiographic patterns:**						
Airspace	Ground Glass (GGO)						
Airspace	Consolidation / Air space (AS)						
Airspace	Mixed GGO/AS						
	Parenchymal micronodules (<7mm)						
Interstitial	Reticular opacities						
Interstitial	Linear opacities or septal lines						
	Peribronchovascular thickening.						
Fibrotic	Honeycombing						
Fibrotic	Traction Bronchiectasis /Bronchiolectases						

SEVERITY: Each zone is to be graded none (0), trace (1), mild (2), moderate (3), severe (4):

0 = No abnormality in that zone

1 = Trace/ minor abnormality in that zone (<5 % area)

2 = Mild abnormality in that zone (5-20%)

3 = moderate or significant abnormality in that zone (>20-50% area)

4 = Severe or predominant abnormality in that zone (> 50%)

- Upper, middle and lower zones separated by aortic arch(upper, middle) and bifurcation of the trachea (middle, lower).
- You can add pleural or central process in comments section.

Supplementary Table 2. Protein biomarkers assessed by Multiplex ELISA

**Cytokines**	IL-1 , IL-1RA, IL-1 , IL-2, IL-2R, IL-4, IL-6, IL-7, IL-8 (CXCL8), IL-10, IL-12p70, IL-13, IL-15, IL-17A, IL-22, TNF-RII, TNF-RI, TNF , IFN , IFN
**Chemokines**	MIG (CXCL9), IP10 (CXCL10), I-TAC (CXCL11)
**Growth Factors**	G-CSF (CSF-3), GM-CSF, EGF, PDGF-BB,VEGF-A
**Remodeling Proteins**	MMP-1,2,3,7,8,9,12,13
